# Quercetin and Sorafenib as a Novel and Effective Couple in Programmed Cell Death Induction in Human Gliomas

**DOI:** 10.1007/s12640-013-9452-x

**Published:** 2013-12-24

**Authors:** Joanna Jakubowicz-Gil, Ewa Langner, Dorota Bądziul, Iwona Wertel, Wojciech Rzeski

**Affiliations:** 1 Department of Comparative Anatomy and Anthropology, Maria Curie-Skłodowska University, Akademicka 19, 20-033 Lublin, Poland; 2 Department of Medical Biology, Institute of Agricultural Medicine, Jaczewskiego 2, 20-950 Lublin, Poland; 3 Department of Immunology and Virology, Maria Curie-Skłodowska University, Akademicka 19, 20-033 Lublin, Poland; 4 1st Department of Gynaecology, University School of Medicine, Staszica 16, 20-081 Lublin, Poland

**Keywords:** Sorafenib, Quercetin, Apoptosis, Autophagy, Gliomas, Heat shock proteins

## Abstract

The aim of the present study was to investigate the effect of sorafenib and quercetin on the induction of apoptosis and autophagy in human anaplastic astrocytoma (MOGGCCM) and glioblastoma multiforme (T98G) cell lines. In MOGGCCM cells, sorafenib initiated mainly apoptosis, mediated by the mitochondrial pathway with mitochondrial membrane permeabilization, cytochrome c release to the cytoplasm, and activation of caspase 9 and 3. Additional incubation with quercetin potentiated the pro-apoptotic properties of sorafenib. In T98G cells, autophagy was observed most frequently after the sorafenib treatment. It was accompanied by increased beclin 1 and LC3II expression. Administration of quercetin after the sorafenib treatment resulted in an increased number of autophagic cells. After simultaneous drug application, the level of autophagy was lower in favour of apoptosis. Inhibition of heat shock proteins expression by specific small interfering RNA significantly increased the sensitivity of both the cell lines to induction of apoptosis, but not autophagy. We demonstrated for the first time that sorafenib and quercetin are very effective programmed cell death inducers in T98G and MOGGCCM cells, especially in cells with blocked expression of heat shock proteins.

## Introduction

Malignant gliomas are the most prevalent primary brain tumours in adults, exhibiting a high rate of cell proliferation and migration activities. The major group is represented by anaplastic astrocytoma (AA, WHO grade III) and glioblastoma multiforme (GBM, WHO grade IV). Despite tremendous efforts in improvement of therapeutics, such as surgery, radiotherapy and chemotherapy, the clinical outcome of gliomas remains dismaying. The median survival in patients with anaplastic astrocytoma is about 30 months, while with glioblastoma multiforme it is less than 15 months under standard care treatment. Therefore, there is an urgent need for new treatments based on a better understanding of the molecular basis of malignant progression of gliomas (Kleihues et al. 2002; Ohgaki and Kleihues 2005; Omuro et al. 2007).

It has been proven that upregulation of the Ras–Raf–MEK–ERK pathway takes part in amplification of mitogenic stimuli and promotion of cellular proliferation of malignant gliomas. Therefore, downregulation of this signal transmission may be a valuable therapy for glioma patients leading to apoptosis or autophagy induction (Lo 2010). A promising candidate for such an action is sorafenib—a small-molecule multikinase inhibitor that was originally developed as an inhibitor of Raf kinase, an essential serine/threonine kinase constituent of the mitogen-activated protein kinase (MAPK) pathway. Intracranial application of sorafenib caused inhibition of cell proliferation, reduction of angiogenesis, and induction of autophagy and apoptosis of glioma cells. Systemic administration of sorafenib was well tolerated and the drug crossed the blood–brain barrier effectively (Hahn and Stadler 2006; Sieglin et al. 2010; Wilhelm et al. 2004). Unfortunately, its use in the clinical treatment of patients with malignant gliomas has yielded disappointing results in some cases (Nabors et al. 2011; Sieglin et al. 2010; Yang et al. 2010). Nevertheless, the ability of sorafenib to inhibit tumour cell proliferation suggests that it may be useful in combination with other therapeutic agents. It is well known that natural active compounds may act in synergy with drugs used in clinical applications. One of them is quercetin (3,3′,4′,5,7-pentahydroxyflavone), a natural flavonoid found in a broad range of fruits and vegetables. It has multiple biological, pharmacological and medical applications and is one of the most potent antioxidants. Quercetin facilitates apoptosis of tumour cells by caspase 3 and caspase 9 activation and cytochrome c release. It is a well-known inhibitor of expression of heat shock proteins (Hsps) (Braganhol et al. 2006; Hosokawa et al. 1992; Ramos 2007; Russo 2007; Schültke et al. 2005).

Therefore, the aim of our studies was to investigate the effect of sorafenib applied alone or in combination with quercetin on induction of apoptosis and autophagy in human glioblastoma multiforme T98G and anaplastic astrocytoma MOGGCCM cell lines. We analysed the typical morphology for programmed cell death as well as the molecular mechanism underlying these processes based on the expression of Hsp27, Hsp72, cytochrome c, LC3, beclin 1, and Ras and Raf proteins and the activity of caspase 3, caspase 8 and caspase 9. Additionally, we studied the sensitivity of MOGGCCM and T98G cell lines with blocked Hsp27 and Hsp72 expression to programmed cell death induction upon combined drug treatment.

## Materials and Methods

### Cells and Culture Conditions

Human glioblastoma multiforme cells (T98G, European Collection of Cell Cultures) and human anaplastic astrocytoma cells (MOGGCCM, European Collection of Cell Cultures) were grown in a 3:1 mixture of Dulbecco’s Modified Eagle Medium (DMEM) and Ham’s nutrient mixture F-12 (Sigma) supplemented with 10 % foetal bovine serum (Sigma), penicillin (100 units/ml) (Sigma) and streptomycin (100 μg/ml) (Sigma). The cultures were kept at 37 °C in humidified atmosphere of 95 % air and 5 % CO_2_.

### Drug Treatment

Sorafenib (Nexavar, BAY 43-9006) at the final concentrations 0.25, 0.5, 0.75, 1, 5 μM and quercetin (3,3′,4′,5,7-pentahydroxyflavone) (Sigma) at the final concentrations 30 μM in the case of MOGGCCM and 50 μM in the case of T98G were used in the experiments. The quercetin concentrations were chosen on the basis of our earlier studies conducted on both the cell lines (Jakubowicz-Gil et al. 2010, 2011, 2013a). The drugs were dissolved in dimethyl sulfoxide (DMSO, Sigma). The final concentration of DMSO in the culture medium did not exceed 0.01 %, which, as indicated in preliminary experiments, did not influence cell viability and the expression of the proteins studied. Two variants of drug treatment were performed. In the first one, the cells were incubated only with sorafenib for 24 or 48 h. In the second one, quercetin and sorafenib were added to the culture medium at the same time and incubated for 24 or 48 h. The control cells were incubated with 0.01 % of DMSO.

### Detection of Apoptosis and Necrosis with Fluorochromes

For identification of apoptosis and necrosis, the cells were stained with fluorescent dyes Hoechst 33342 (Sigma) and propidium iodide (Sigma), respectively (Jankowska et al. 1997). The morphological analysis was performed under a fluorescence microscope (Nikon E—800). Cells exhibiting blue fluorescent nuclei (fragmented and/or with condensed chromatin) were interpreted as apoptotic. Cells exhibiting pink fluorescent nuclei were interpreted as necrotic. At least 1,000 cells in randomly selected microscopic fields were counted under the microscope. Each experiment was performed in triplicate.

### Detection of Acidic Vesicular Organelles with Acridine Orange

Autophagy is a process of sequestrating cytoplasmic proteins into the lytic compartment and is characterized by formation and promotion of acidic vesicular organelles (AVOs). Vital staining with 1 μg/ml of acridine orange was performed for 15 min to detect AVOs in cells treated with quercetin and/or sorafenib (Takeuchi et al. 2005). Typical acridine orange-positive cells exhibiting granular discretion of AVOs in the cytoplasm were interpreted as autophagic. The morphological analysis was performed under a fluorescence microscope (Nikon E—800). At least 1,000 cells in randomly selected microscopic fields were counted. Each experiment was performed in triplicate. The percentage of autophagic cells was calculated as the number of cells with AVOs versus the total number of stained cells.

### Detection of Apoptosis and Necrosis by Flow Cytometry

The Annexin V-FITC apoptosis detection kit (Sigma) was used for detection of apoptosis and necrosis by flow cytometry. The samples were prepared according to the manufacturer’s protocol. Briefly, the control and drug-treated cells were incubated with 5 μl of Annexin V-FITC and 10 μl of propidium iodide for 10 min. Immediately after staining, the cells were analysed with the FacsCanto instrument (Becton–Dickinson, San Jose, CA, USA). In total, 30,000 events were acquired and analysed using FacsDiva software. Cells that were in the early apoptotic process were stained only by Annexin V-FITC; late apoptotic cells were stained by both the fluorochromes. Cells stained only by propidium iodide were interpreted as necrotic. Live cells showed no staining by either propidium iodide or Annexin V-FITC. Each experiment was performed in triplicate.

### Mitochondrial membrane potential

Fluorochrome 3,3′-dihexyloxacarbocyanine iodide [DiOC_6_(3)] was used for studying the changes in the mitochondrial membrane potential (∆*ψ*_m_) in cells incubated with quercetin and sorafenib. At low concentrations, the stain accumulates in mitochondria. Loss of DiOC_6_(3) staining indicates disruption of the mitochondrial inner transmembrane potential (Kim et al. 2008). The control and drug-treated cells were incubated with fluorochrome at the final concentration 50 nM for 20 min at 37 °C in the dark, washed three times with phosphate buffered saline (PBS), and analysed with the FacsCanto instrument (Becton–Dickinson, San Jose, CA, USA). In total, 30,000 events were acquired and analysed using FacsDiva software. Each experiment was performed in triplicate. The results were expressed as relative intensity of DiOC_6_(3) brightness.

### Mitochondrial Fraction Isolation

A commercial isolation kit was used (Sigma) for isolation of mitochondria. The samples were prepared according to the manufacturer’s protocol. Briefly, after quercetin or/and sorafenib treatment at the density of about 2 × 10^7^, the cells were trypsinised, centrifuged for 5 min (600×*g*) and washed twice with PBS. The cell pellet was resuspended in lysis buffer (Sigma), incubated for 5 min on ice and centrifuged for 10 min (600×*g*). The supernatant was transferred into a fresh tube and centrifuged at 11,000×*g* for 10 min. The pellet was resuspended in cell lysis buffer and used for electrophoresis.

### Isolation of the Cytosolic Fraction

After the quercetin and/or sorafenib treatment, the cells were lysed in hot lauryl sulphate (SDS)-loading buffer (125 mM Tris–HCl pH 6.8; 4 % SDS; 10 % glycerol; 100 mM dithiothreitol), boiled in water bath for 10 min and centrifuged at 10,000×*g* for 10 min; next, the supernatants were collected. The protein concentration was determined by the Bradford method (Bradford 1976) and samples of the supernatants containing 80 μg of proteins were used for electrophoresis.

### Immunoblotting

The cytoplasmic and mitochondrial samples were separated by 10 % SDS-polyacrylamide gel electrophoresis (Laemmli 1970) and subsequently transferred onto an Immmobilon P membrane (Sigma). Following the transfer, the membrane was blocked with 3 % low-fat milk in PBS for 1 h and incubated overnight with a mouse anti-Hsp72 monoclonal antibody (SPA 810, StressGen) at the concentration 0.2 μg/ml, anti-Hsp27 (SPA 800, StressGen) at the concentration 0.1 μg/ml, rabbit anti-LC3 (Sigma) at the concentration 2 μg/ml, anti-beclin 1 antibody (Sigma) at the concentration 3 μg/ml, anti-Ras (Santa Cruz Biotechnology) at the concentration 0.5 μg/ml, anti-Raf (Santa Cruz Biotechnology) at the concentration 0.5 μg/ml, and sheep anti-cytochrome c antibody (Sigma) at the concentration 0.2 μg/ml. The membranes were washed three times with PBS containing 0.05 % Triton X-100 (Sigma) for 10 min and incubated for 2 h with alkaline phosphatase-conjugated goat anti-mouse, anti-sheep or anti-rabbit secondary antibodies (Sigma). The membranes were visualised with an alkaline phosphatase substrate (5-bromo-4-chloro-3-indolylphosphate and nitro-blue tetrazolium, Sigma) in a colour development buffer *N*,*N*-dimethylformamide (DMF, Sigma). The data were normalised relative to β-actin (detected by mouse anti-β-actin antibodies at the concentration 0.5 μg/ml, Sigma).

The levels of protein expression were determined using the Bio-Profil Bio-1D Windows Application V.99.03 programme. The value was expressed as relative intensity of band thickness, width and colour depth. Three independent experiments were performed.

### Caspase Activity Assay

Caspases are cysteine proteases that exist in normal conditions as inactive pro-forms or zymogens. They are cleaved to the active form following induction of apoptosis. The activity of caspases 3, 8 and 9 was estimated using a SensoLyte^®^AMC Caspase Substrate Sampler Kit (AnaSpec) in the control and drug-treated cells. Sample preparation and the enzymatic reaction were performed according to the manufacturer’s protocol. The fluorescence of 7-aminocoumarin (AMC) was monitored at *E*_x_/*E*_m_ = 354/442 nm in 96-well black microplates using a 2030 Multilabel Reader Victor^TM^x4 (Perkin Elmer).

### Cell Transfection

The MOGGCCM and T98G cells at a density of 2 × 10^5^ were seeded in a six-well tissue culture plate and incubated at 37 °C in a CO_2_ incubator for 24 h until confluence reached 60–80 %. The cells were washed with a 3:1 DMEM:Ham’s F-12 mixture without serum and antibiotics, and aspirated. For each transfection, probes containing 2 μl of a specific anti-Hsp27 or anti-Hsp72 small interfering RNA (siRNA) duplex (Santa Cruz Biotech) and 2 μl of Transfection Reagent (Santa Cruz Biotech) were prepared, overlaid onto washed cells and incubated for 5 h at 37 °C in a CO_2_ incubator. After this time, the medium was enriched with a growth medium containing 20 % of foetal bovine serum and 200 μg/ml of antibiotics without removing the transfection mixture and the cells were incubated for additional 18 h. Then the medium was replaced by a fresh normal growth medium and the transfected cells were used for further experiments. The effectiveness of gene silencing was analysed at the protein level by the immunoblotting technique.

### Indirect Immunofluorescence

After the treatment with quercetin and/or sorafenib, the cells were washed three times with PBS and fixed in 3.7 % paraformaldehyde (Sigma) in PBS for 10 min. After extensive washing with PBS, the cells were treated with 0.2 % Triton X-100 (Sigma) for 7 min and then washed three times with PBS, all at room temperature. Subsequently, a blocking step of 30-min incubation in 5 % low-fat milk at room temperature was included. Next, the cells were incubated with rabbit antibody anti-beclin 1(Sigma) at a concentration of 30 μg/ml. The primary antibodies were detected with fluorescein (FITC)-conjugated anti-rabbit antibodies (Sigma).

Protein localisation in the cells was analysed using the scanning head PASCAL5 (Zeiss). The fluorescent channel was *λ* = 488 nm. Three independent experiments were performed. Over 100 cells were analysed in each experimental variant.

### Statistical Analysis

The data are presented as mean ± standard deviation (SD). The statistical evaluation was performed with a one-way Anova test followed by Dunnett’s multiple comparison test. *P* < 0.05 was taken as the criterion of significance.

## Results

### Sensitivity of T98G and MOGGCCM Cells to Sorafenib Treatment

**Fig. 1:**
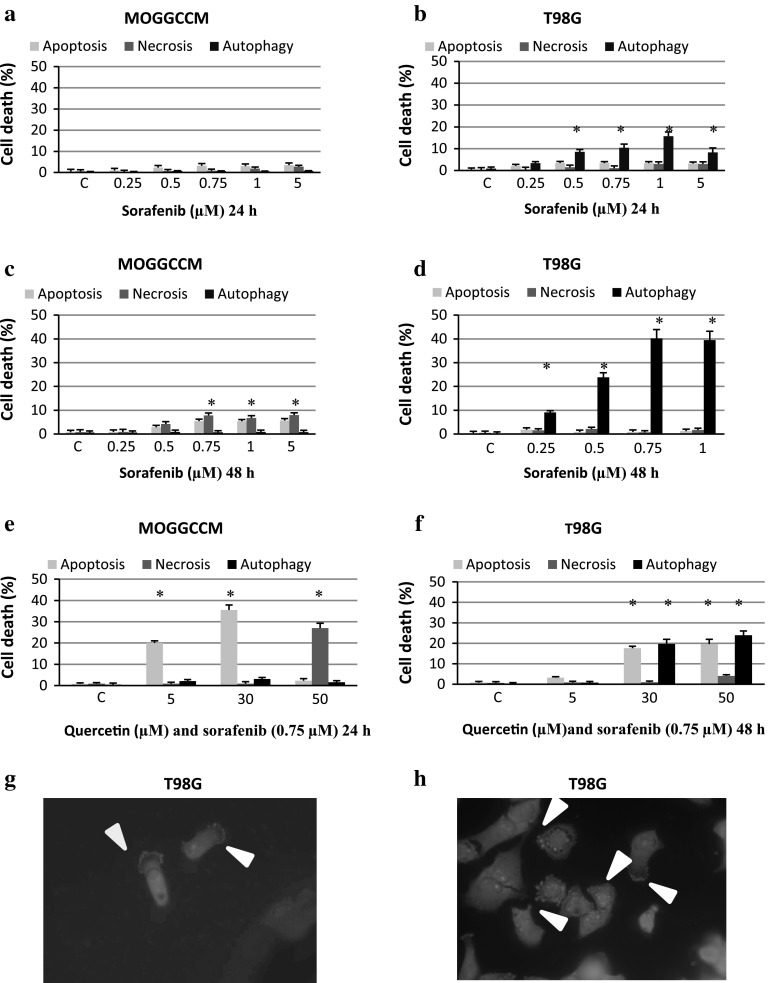
The level of apoptosis, necrosis and autophagy induction in the anaplastic astrocytoma MOGGCCM (**a**, **c**, **e**) and the glioblastoma multiforme T98G (**b**, **d**, **f**–**h**) cell lines treated only with sorafenib (0–5 μM) for 24 h (**a**, **b**) or 48 h (**c**, **d**) and sorafenib in combination with quercetin (**e**, **f**) stained with Hoechst 33342, propidium iodide and acridine orange, C-control cells (without sorafenib or quercetin), *P < 0.05 compared to control g—localisation of the autophagy marker protein beclin 1 in T98G cells after treatment with sorafenib (0.75 μM) for 48 h (pointed by *arrows*) h—autophagic vacuoles in T98G cells stained with acridine orange after 48-h-long incubation with sorafenib (0.75 μM) (pointed by *arrows*)

To estimate the sensitivity of T98G and MOGGCCM cells to the sorafenib treatment, a staining method with dyes specific for apoptosis, necrosis and autophagy; i.e. Hoechst 33342, propidium iodide and acridine orange, respectively, was employed (Fig. 1). Microscopic observations revealed that sorafenib applied at the concentrations 0–5 μM to the MOGGCCM culture medium for 24 h (Fig. 1a) had no considerable effect on cell death. A significant increase in the number of apoptotic cells was observed after 48 h of incubation, reaching a maximum (5.6 %) at the concentration 5 μM (Fig. 1c). Besides apoptosis, sorafenib initiated necrosis at a level exceeding the percentage of apoptotic cells. In the case of T98G, sorafenib applied to the culture medium for 24 (Fig. 1b) and 48 h (Fig. 1d, h) appeared to be a potent autophagy inducer. In the case of 24-h long incubation, sorafenib at the concentration 1 μM initiated the process in 16 % of the cells. The longer incubation time was even more effective. About 40 % of cells treated with 0.75 or 1 μM of sorafenib exhibited bright red vesicles at the edge of cells observed after staining with acridine orange (Fig. 1h). The indirect immunofluorescence technique revealed that the autophagy marker beclin1 was localised at these vesicles (Fig. 1g). The drug had no significant effect on induction of apoptosis and necrosis in T98G cells.

### Sensitivity of T98G and MOGGCCM Cells to Combined Sorafenib and Quercetin Treatment

Based on the results described earlier, to examine the combined effect of sorafenib with quercetin, the drug at the concentration 0.75 μM applied to MOGGCCM cells for 24 h and T98G cells for 48 h was chosen. The incubation of T98G cells with sorafenib at the concentration 0.75 μM with increasing quercetin concentrations (0–50 μM) for 48 h revealed that such a combination mainly induced programmed cell death. When the cells were treated simultaneously with the combination of 50 μM of quercetin and 0.75 μM of sorafenib, apoptosis was observed in 20 % of the cells, which was accompanied by autophagy in 25 % of the cells (Fig. 1f). When the MOGGCCM cells were incubated with sorafenib (0.75) and quercetin (30 μM) for 24 h, apoptosis was the most frequent (35 %) (Fig. 1e). Co-incubation with sorafenib and flavonoid at the concentration 50 μM resulted in severe necrosis. The level of autophagy was not significant.

### Evaluation of Cell Death and Mitochondrial Transmembrane Potential by Flow Cytometry

To confirm the reliability of the results obtained through the microscopic observations, T98G cells treated with quercetin (50 μM) and sorafenib (0.75 μM) for 48 h and MOGGCCM cells treated with quercetin (30 μM) and sorafenib (0.75 μM) for 24 h separately as well as in combination of both the drugs were analysed by flow cytometry according to the type of cell death (apoptosis or necrosis) and the mitochondrial membrane potential (Balasubramanian and Schroit 2003; Ghobrial et al. 2005).

The incubation of the cells with quercetin confirmed that the flavonoid induced mainly necrosis in the MOGGCCM (Table 1a) and had no such effect on the T98G cells (Table 1b). Sorafenib alone induced mainly late apoptosis in both the cell lines at the level of 6 %. When quercetin and sorafenib were added to the culture medium together, late apoptosis was frequently observed in the MOGGCCM cells (6.9 %). In the case of the T98G cells, early apoptosis was predominant and reached about 6.3 %.

**Table 1:** The effect of quercetin (Q) and sorafenib (S) on apoptosis and necrosis induction in MOGGCCM (a) and T98G (b) cells analysed by flow cytometry with Annexin V-FITC detection kit

	Necrosis (%)	Early apoptosis (%)	Late apoptosis (%)
a			
Control	0.2 ± 0.09	0.2 ± 0.2	0.2 ± 0.07
Q (30 μM)	48.8 ± 5.3*	0.5 ± 0.14	0.2 ± 0.07
S (0.75 μM)	0.7 ± 0.4	0.3 ± 0.07	6.7 ± 0.35*
SQ	0.5 ± 0.07	1.3 ± 0.77	6.9 ± 0.29*
b			
Control	0.3 ± 0.07	0.2 ± 0.07	0.2 ± 0.07
Q (50 μM)	2.7 ± 0.4	8.2 ± 1.2*	8.6 ± 0.07*
S (0.75 μM)	0.1 ± 0.07	2.7 ± 0.96	6.3 ± 0.29*
SQ	0.3 ± 0.5	6.3 ± 0.77*	4.8 ± 0.4*

* *P* < 0.05

**Fig. 2:**
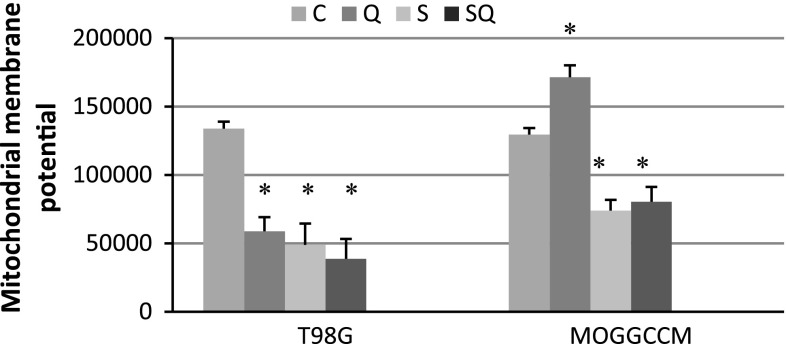
The mitochondrial membrane potential in MOGGCCM and T98G cells stained with DiOC_6_(3), incubated separately with quercetin (Q) and sorafenib (S) or in combination of both the drugs (SQ) for 24 h (MOGGCCM) or 48 h (T98G) and analysed by flow cytometry, *C* control cells, *P < 0.05 compared to control

It is known that a decreased mitochondrial membrane potential is a good indicator of apoptotic cell death (Ghobrial et al. 2005). In our experiments, both quercetin and sorafenib applied alone or in combination decreased this potential in the T98G and MOGGCCM cells (Fig. 2). In the glioblastoma multiforme cells, the lowermost value was observed after simultaneous drug treatment. In the case of MOGGCCM cells, sorafenib alone or in combination with quercetin decreased the mitochondrial membrane potential most effectively, in comparison to the control. An increase in this parameter was observed only after incubation with quercetin.

### The Effect of Sorafenib and Quercetin on Expression of Marker Proteins

**Fig. 3:**
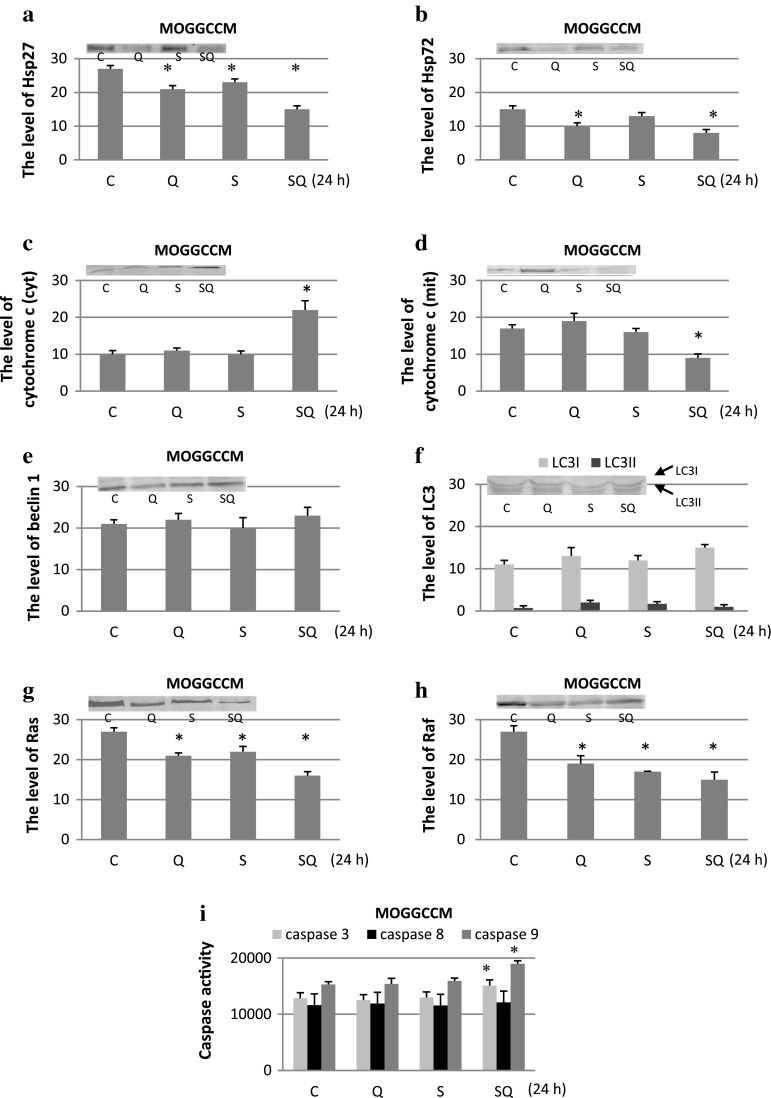
The level of Hsp27 (**a**), Hsp72 (**b**), cytochrome *c* (**c** cytoplasmic, **d** mitochondrial fraction), beclin 1 (**e**), LC3 (**f**), Ras (**g**) and Raf (**h**) expression with representative blots and the activity of caspase 3, 8, 9 (i) after sorafenib (S) and quercetin (Q) treatment for 24 h in MOGGCCM. The data were normalised relative to β-actin (not shown). *C* control cells, *SQ* simultaneous drug treatment, * *P* < 0.05 compared to control

**Fig. 4:**
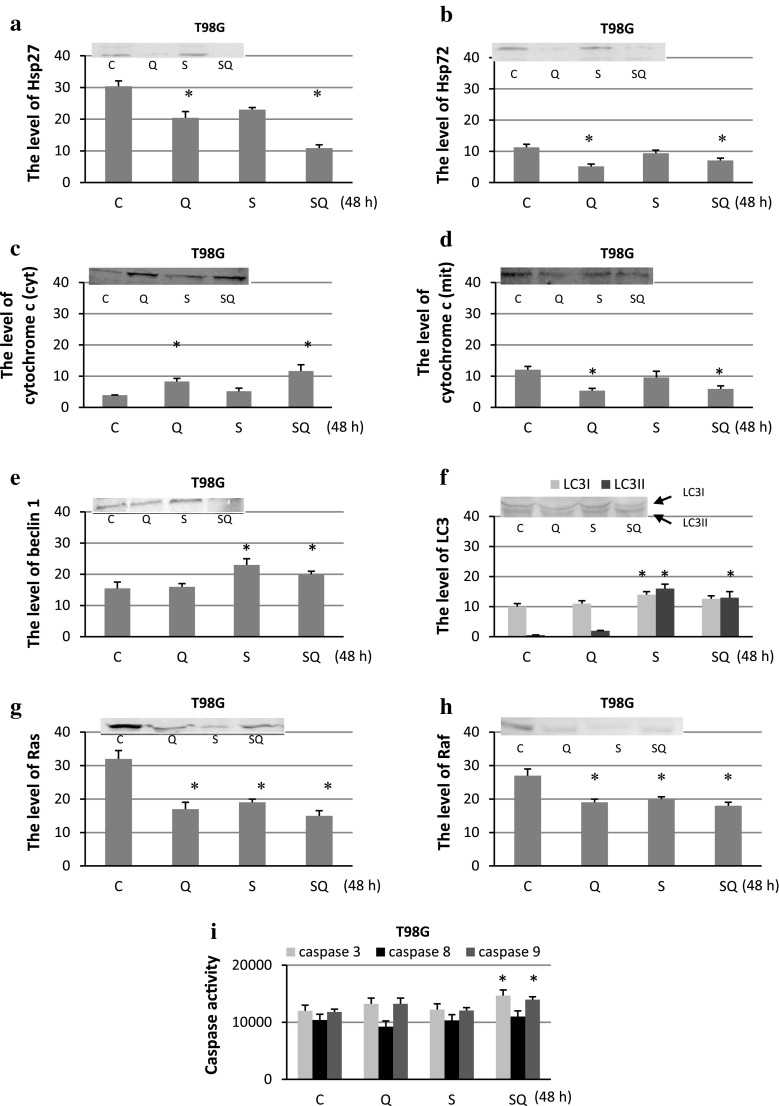
The level of Hsp27 (**a**), Hsp72 (**b**), cytochrome *c* (**c** cytoplasmic, **d** mitochondrial fraction), beclin 1 (**e**), LC3 (**f**), Ras (**g**) and Raf (**h**) expression with representative blots and the activity of caspase 3, 8, 9 (*i*) after sorafenib (S) and quercetin (Q) treatment for 48 h in T98G. The data were normalised relative to β-actin (not shown). *C* control cells, *SQ* simultaneous drug treatment, *P < 0.05 compared to control

Using the immunoblot technique, we studied the effect of sorafenib and quercetin, applied separately or in combinations, on the level of the pro-apoptotic cytochrome c expression (in the cytoplasmic and mitochondrial fraction), anti-apoptotic molecular chaperones Hsp27 and Hsp72, pro-survival Ras, Raf, and pro-autophagic beclin1and LC3 in the MOGGCCM (Fig. 3) and T98G (Fig. 4) cells. Additionally, we analysed fluorimetrically the activity of caspases 3, 8 and 9.

Quantitative and qualitative analyses of the immunoblots revealed that quercetin and sorafenib applied alone or in combination inhibited the expression of Hsp72, Hsp27, Ras and Raf in all the experimental variants in both the cell lines. The drugs were also very effective in decreasing the level of cytochrome *c* in the mitochondrial fraction, which was accompanied by increased accumulation of the protein in the cytoplasm. In the case of beclin 1, quercetin and sorafenib had no significant effect on the protein expression in the MOGGCCM cells and its level was similar to the control one in all the experimental variants. In the T98G cells, overexpression of beclin 1 was observed after separate sorafenib treatment and after sorafenib with quercetin. In other experimental variants, the level of beclin 1 was similar to the control. Conversion of LC3I into its smaller form LC3II is the hallmark of autophagy. Similar to beclin1, increased level of LC3II was observed only in T98G cells after separate sorafenib treatment and in combination with quercetin. In the case of caspases, quercetin and sorafenib applied in combination (but not in the separate application) increased the activity of caspase 3 and caspase 9 in the MOGGCCM cells. In the T98G cells, elevated activity of the enzymes was observed after separate quercetin treatment and when both the drugs were added at the same time. Sorafenib and quercetin applied alone or in combination had no effect on caspase 8 activity in the MOGGCCM and T98G cells.

### Blocking the Hsp27 and Hsp72 Expression in T98G and MOGGCCM Cells

**Fig. 5:**
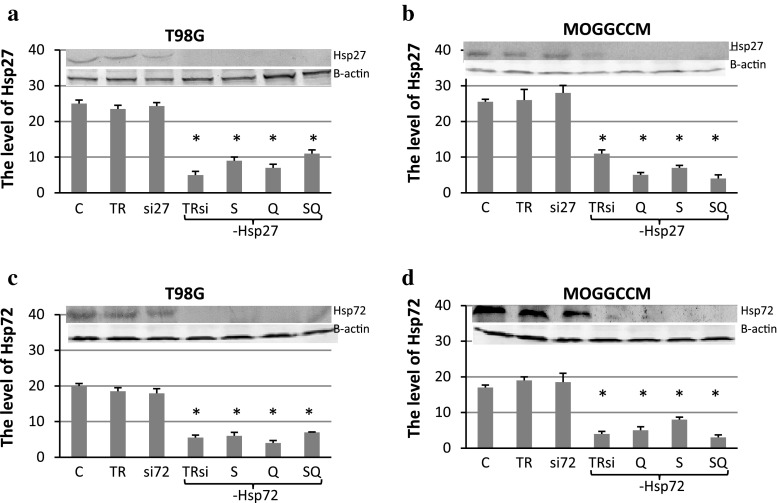
The level of Hsp27 and Hsp72 expression in T98G (**a**, **c**) and MOGGCCM (**b**, **d**) cells after transfection with specific anti-Hsp27 (**a**, **b**) or anti-Hsp72 (**c**, **d**) siRNA (si27 and si72, respectively) and subsequent quercetin (Q) and sorafenib (S) treatment, estimated by immunoblotting. T98G cells were incubated with 50 μM of quercetin and 0.75 μM sorafenib, while MOGGCCM cells with 30 μM (Q30) of quercetin and 0.75 μM of sorafenib. Protein level was normalised according to β-actin expression. *C* control, *TR* transfection reagent, *TRsi* transfection reagent with specific siRNA, *SQ* simultaneous quercetin and sorafenib treatment, *P < 0.05 compared to control

To block the expression of Hsp27 and Hsp72, the T98G and MOGGCCM cells were transfected with specific siRNA. Western blot analysis revealed that the silencing was very effective at the protein level (Fig. 5) and no expression of Hsps was observed even after subsequent quercetin and/or sorafenib treatment. Incubation of the cells either with only the transfection reagent or with only siRNAs had no effect on the expression of heat shock proteins.

### Apoptosis, Autophagy and Necrosis Induction in Transfected Cells

**Fig. 6:**
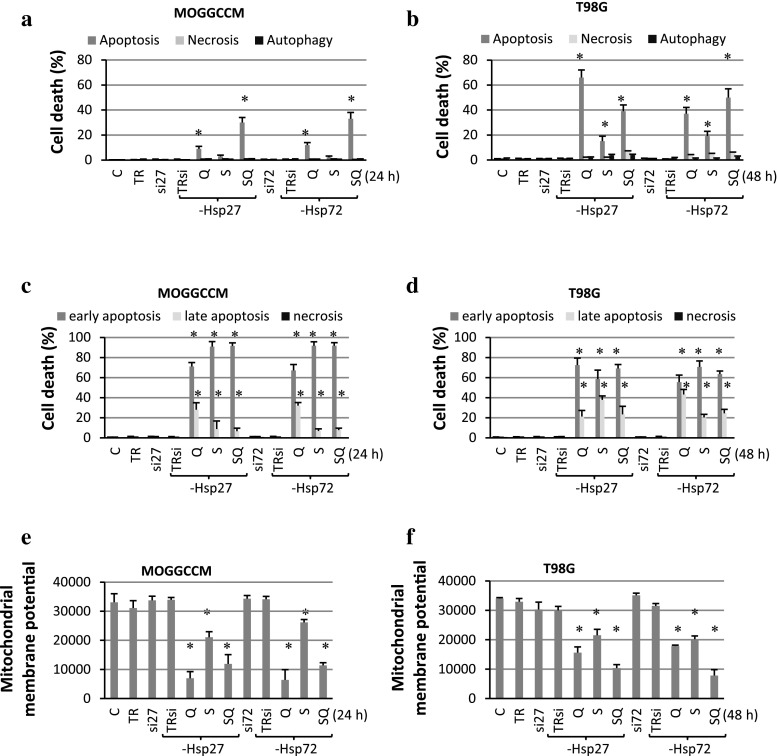
The effect of quercetin (Q) and sorafenib (S) on apoptosis, necrosis and autophagy induction in MOGGCCM (**a**, **c**, **e**) and T98G (**b**, **d**, **f**) cells transfected with specific siRNA anti-Hsp27 (si27) and anti-Hsp72 (si72). *C* control, *TR* transfection reagent, **a**, **b** cell death estimated by microscopic observation of cells stained with Hoechst 33342, propidium iodide, acridine orange, **c**, **d** apoptosis and necrosis induction estimated by flow cytometry with the Annexin V-FITC detection kit, **e**, **f** the mitochondrial membrane potential studied by flow cytometry after staining with DiOC_6_(3), *P < 0.05 compared to control

The effect of sorafenib and quercetin on cell death induction in the T98G and MOGGCCM (Fig. 6) cells with blocked Hsp expression was studied by means of microscopic observations (Fig. 6a, b) and flow cytometry (Fig. 6c, d). Both the methods revealed that quercetin and sorafenib were extremely effective in cell death induction in transfected MOGGCCM and T98G cells, especially when both the drugs were added to the culture medium simultaneously.

Microscopic observations of T98G cells with diminished Hsp27 expression showed over 60 % of apoptotic cells after the quercetin treatment, 15 % after the sorafenib incubation and 40 % after the simultaneous drug application. In the case of reduced Hsp72 expression, sorafenib applied in combination with the flavonoid appeared to be the most effective in apoptosis induction, as it initiated programmed cell death in about 50 % of cells. Sorafenib itself appeared to be the least effective in induction of apoptosis (19 %) but still the result was significant. In the MOGGCCM cells, blocking of the Hsp27 or Hsp72 expression resulted in an increased number of apoptotic cells after the simultaneous sorafenib and quercetin treatment (about 30 %). Quercetin was less effective but still the effect was significant. Interestingly, blocking of the Hsp27 or Hsp72 expression did not increase the sensitivity of the cell line to apoptosis induction after the sorafenib treatment.

In comparison to the microscopic observations, flow cytometry appeared to be a more sensitive method for detection of apoptosis in MOGGCCM and T98G cells transfected with specific siRNA. Apoptosis was observed in over 90 % of the cells, irrespective of the type of cells, type of blocked protein and subsequent drug treatment. The method also allowed distinguishing between early and late apoptosis. As shown in Fig. 6c and d, apoptosis after the quercetin and/or sorafenib treatment was detected mainly in the early stage of the process in both the cell lines.

Our results indicate that inhibition of Hsp72 or Hsp27 expression had no significant effect on necrosis or autophagy induction upon the quercetin and sorafenib treatment.

### The Effect of Quercetin and Sorafenib on the Mitochondrial Membrane Potential in Transfected Cells

The flow cytometry analysis of DiOC_6_(3)-stained MOGGCCM and T98G cells with blocked Hsp27 and Hsp72 expression revealed that quercetin and sorafenib applied alone or in combination significantly decreased the mitochondrial membrane potential. As shown in Fig. 6e, such an inhibitory effect in the MOGGCCM cells was the most efficient after the quercetin treatment, while in the T98G cells (Fig. 6f)—after the simultaneous drug incubation.

### The Effect of Sorafenib and Quercetin on Marker Protein Expression in Transfected Cells

**Fig. 7:**
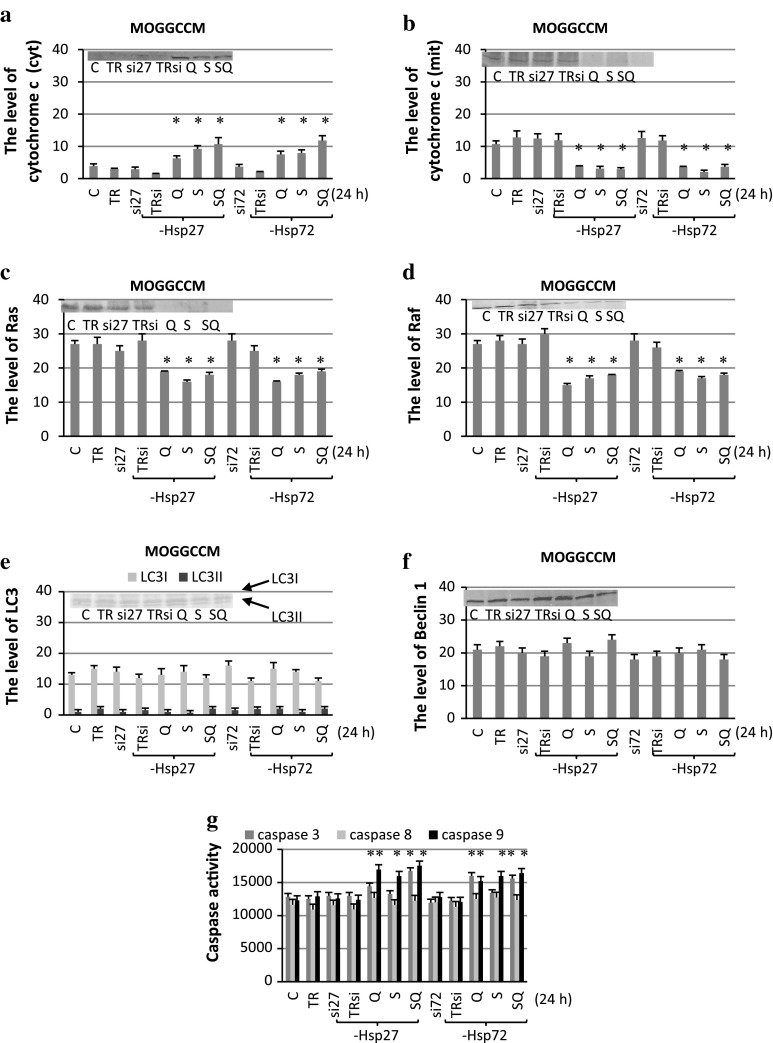
The level of cytochrome *c* (**a** cytoplasmic, **b** mitochondrial fraction), Ras (**c**), Raf (**d**), LC3 (**e**) and beclin 1 (**f**) expression with representative blots and the activity of caspase 3, 8, 9 (**f**) after sorafenib (S) and quercetin (Q) treatment for 24 h in MOGGCCM cells transfected with specific siRNA anti-Hsp27 (si27) and anti-Hsp72 (si72). The data were normalised relative to β-actin (not shown). *C* control cells, *SQ* simultaneous drug treatment, *si27 or si72* specific siRNA blocking Hsp27 or Hsp72 expression, *TR* transfection reagent, *TRsi* transfection reagent with specific siRNA *P < 0.05 compared to control

**Fig. 8:**
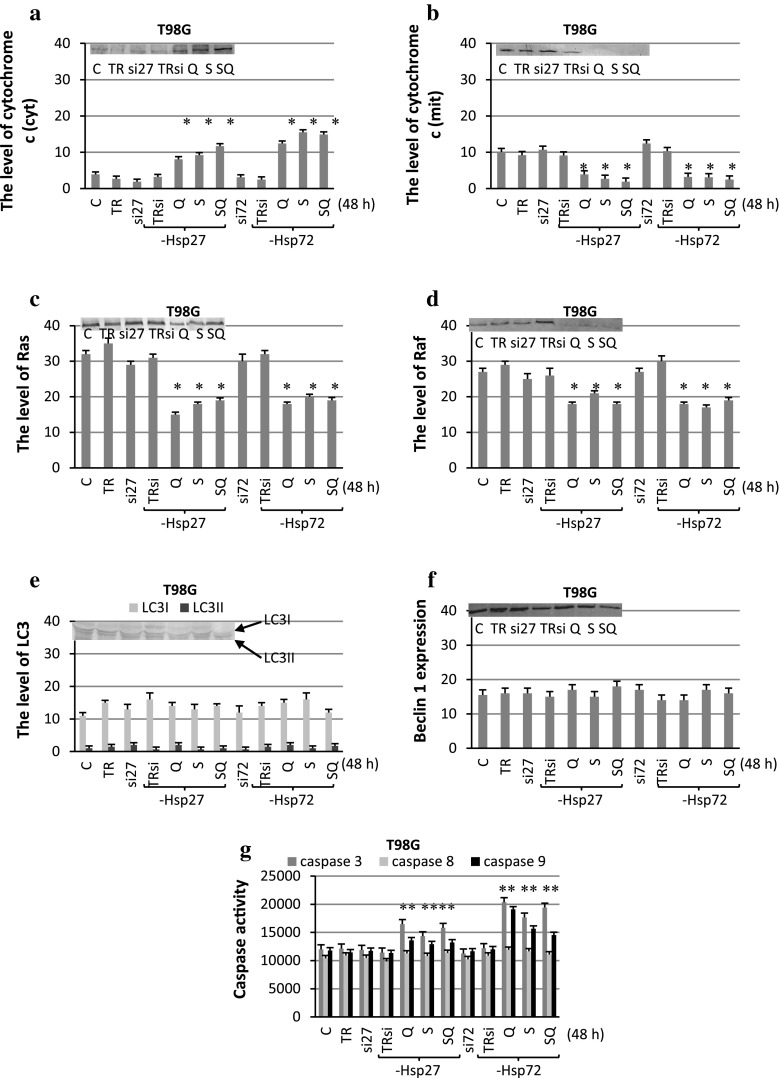
The level of cytochrome c (**a** cytoplasmic, **b** mitochondrial fraction), Ras (**c**), Raf (**d**), LC3 (**e**) and beclin 1 (**f**) expression with representative blots and the activity of caspase 3, 8, 9 (**g**) after sorafenib (S) and quercetin (Q) treatment for 48 h in T98G cells transfected with specific siRNA anti-Hsp27 (si27) and anti-Hsp72 (si72). The data were normalised relative to β-actin (not shown). *C* control cells, *SQ* simultaneous drug treatment, *si27 or si72* specific siRNA blocking Hsp27 or Hsp72 expression, *TR* transfection reagent, *TRsi* transfection reagent with specific siRNA, *P < 0.05 compared to control

Quantitative and qualitative analyses of immunoblots with proteins isolated from the MOGGCCM (Fig. 7) and T98G (Fig. 8) cells with blocked Hsp27 or Hsp72 expression revealed that quercetin and sorafenib applied alone or in combination increased the level of pro-apoptotic cytochrome c in the cytoplasm, which was accompanied by a decreased level of the protein in the mitochondrion. Neither of the drugs had an effect on the beclin 1 expression and the level of the protein was similar to the control. No conversion of LC3I into its smaller form LC3II was also observed. Quercetin and sorafenib applied alone or in combination inhibited the expression of Ras and Raf in both the cell lines. In the case of caspases, both the drugs applied alone or in combination increased the activity of capase 3 and caspase 9 in T98G and MOGGCCM cells with blocked Hsp expression. Sorafenib and quercetin did not change the activity of caspase 8 in either cell line.

## Discussion

In malignant gliomas, several mechanisms responsible for programmed cell death induction, like apoptosis or autophagy, are blocked while molecular chaperones promoting cell survival are overexpressed (Ghobrial et al. 2005; Ohgaki and Kleihues 2005; Omuro et al. 2007). Recent publications indicate that a majority of gliomas display upregulated Raf kinase (the first effector kinase downstream of Ras), which is essential for activation of the mitogen-activated protein pathway Ras–Raf–MEK–ERK. The upregulation of this signalling cascade has been proven to take part in amplification of mitogenic stimuli and promotion of cellular proliferation of gliomas (Lo 2010). Such observations provide a rationale for attempting to disrupt this pathway as a good candidate in anticancer strategy. Recently, a number of specific inhibitors have been developed. Among these, sorafenib seems to have a pivotal role. It has been demonstrated in various tumour cell lines that sorafenib induced mitochondrial damage manifested by cytochrome c release into the cytosol, caspase 9 and 3 activation, and in consequence, apoptosis mediated through an intrinsic pathway (Huang et al. 2010; Rahmani et al. 2007; Yu et al. 2006). On the other hand, other experiments showed that apoptosis after sorafenib treatment was correlated with an external pathway with caspase 8 activation (Park et al. 2010). There are also some reports indicating that sorafenib also induced autophagy (Sieglin et al. 2010). Considering such multiple effects of sorafenib on induction of programmed cell death and different mechanisms activated within cells, we decided to evaluate the type of cell death that will be predominant in human glioblastoma multiforme (T98G) and anaplastic astrocytoma (MOGGCCM) cells. Our experiments showed that in anaplastic astrocytoma, the drug induced mainly apoptosis, but at a level only slightly higher than that observed in non-treated, control cells. The process was initiated through a mitochondrial pathway. In the case of glioblastoma multiforme cells, sorafenib appeared to be a very effective autophagy inducer. At the molecular level, this was correlated with the increased conversion of LC3I form of LC3 into LC3II—the first mammalian protein indentified that specifically associates with autophagosome membranes. Therefore, the cleavage of LC3I into LC3II is the hallmark of autophagy (Kabeya et al. 2000). Increased beclin 1 expression, a critical component involved in autophagosome formation in the early stage of autophagy, was also observed. It has also been shown that activation of caspase 3 inhibits autophagosome formation and may block beclin 1 activity or even cleave the protein (Luo and Rubinsztein 2010; Pirtoli et al. 2009; Wang 2008; Wirawan et al. 2010). In the T98G cells, the activity of caspase 3 was not significant, in contrast to the anaplastic astrocytoma cells, where the activity of the enzyme was increased.

The very interesting morphology of autophagic T98G cells should be emphasized. Death vesicles were concentrated at the edges of cells but not dispersed throughout the cytoplasm. The authenticity of the peripheral vacuoles as autophagic ones was confirmed by localisation of beclin 1 within them. The cause of such an unusual distribution is unknown.

Natural bioactive compounds may act in synergy with drugs used in anticancer therapy (Braganhol et al. 2006; Jakubowicz-Gil et al. 2010, 2011, 2013a; Ramos 2007; Russo 2007; Schültke et al. 2005). In the present pioneer study, the use of the combination of sorafenib with quercetin resulted in an effective cell type-specific induction of apoptosis and autophagy. After the treatment with both the drugs, apoptosis was most frequent in the anaplastic astrocytoma cells. The percentage of dead cells was higher than that observed after the single sorafenib applications. At the molecular level, apoptosis was mediated by an intrinsic pathway. In the case of T98G cells treated with sorafenib and quercetin, the level of dead cells was comparable with that observed after the separate sorafenib treatment; and simultaneous incubation with both the drugs induced both apoptosis and autophagy. Keeping in mind that quercetin induces mainly apoptosis while sorafenib mainly autophagy, it seems that these drugs initiated different pathways in the glioblastoma multiforme cell line. As described earlier, the balance between caspase 3 and beclin 1 may have a critical role in such diversity.

In our study, sorafenib as well as quercetin applied alone or in combination decreased the level of Ras and Raf expression in the MOGGCCM and T98G cells, thereby upregulating the Ras–Raf–MEK–ERK pathway. The level of inhibition was similar in both the cell lines, irrespective of the drug precedence and concentration. This indicates that decreased signal transmission through the Ras–Raf–MEK–ERK cascade increases the sensitivity of glioma cells to death upon sorafenib and quercetin treatment and stimulates apoptosis and autophagy induction with the same effect.

Heat shock proteins are molecular chaperones whose activity protects cells against death. They are engaged in preventing cytochrome c release, mitochondrial membrane permeabilization, maturation of procaspase 9 and caspase 3 activation. Depletion of Hsp27 and Hsp72 severely decreases degradation of misfolded proteins (Stetler et al. 2010; Turturici et al. 2011). In cancer cells, where the level of heat shock proteins is abnormally high, these molecular chaperones act as negative prognostic markers. It was also observed that the expression of the proteins rose with the grade of the tumour and that they might participate in oncogenesis. Therefore, inhibition of heat shock protein expression has become a novel strategy for cancer therapy (Garrido et al. 2006; Graner et al. 2007; Jakubowicz-Gil et al. 2013b; Sreedhar and Csermley 2007). In our experiments, both sorafenib and quercetin partially inhibited Hsp27 and Hsp72 expression in the T98G and MOGGCCM cells. Additional block of their expression by specific siRNA showed very good predisposition of the combination of the drugs to induce apoptosis in transfected cells. This type of programmed cell death was detected in about 90 % of the cell population, where most cells were in the early stage of the process. As revealed by the molecular analysis, apoptosis in the transfected cells was mediated by an intrinsic signal.

Blocking of Hsp27 and Hsp72 expression in the T98G and MOGGCCM cells did not increase sensitivity to autophagy induction upon sorafenib and quercetin treatment. It is known that therapeutically increased autophagy could represent an alternative way to destroy cancer cells. This would be beneficial in the light of recent investigations, as gliomas naturally resist apoptosis. On the other hand, many studies demonstrate that autophagy represents a protective mechanism helping cancer cells to get rid of toxic particles and, in consequence, to increase their survival. In the light of these observations, redirecting cancer cells to enter the apoptotic pathway would be beneficial (Garcia-Arencibia et al. 2010; Lefranc and Kiss 2006; Lin et al. 2012). Eliminating Hsp expression by siRNA significantly increased the sensitivity of the T98G cell line to induction of apoptosis but not autophagy. This may be explained by the fact that Hsps are very effective caspase 3 inhibitors. Elimination of these molecular chaperones from cells does not result in blocking caspase 3 activity, which in turn may decrease the level of beclin 1 and block autophagy in consequence.

In summary, we demonstrated for the first time that sorafenib applied in combination with quercetin is a very potent programmed cell inducer in glioma multiforme T98G and anaplastic astrocytoma MOGGCCM cells but the type of death, apoptosis or autophagy, is cell type and drug precedence specific. Our results indicate that blocking of Hsp27 and Hsp72 expression makes T98G and MOGGCCM cells extremely vulnerable to apoptosis induction upon sorafenib and quercetin treatment and that programmed cell death is initiated by an internal signal. They also confirm that molecular chaperones are responsible for glioma cell resistance to programmed cell death and that the natural bioactive compound acts in synergy with anticancer drugs. We demonstrated that sorafenib administered with quercetin seems to be a potent and promising combination that might be useful in glioma therapy.
